# What about India Rubber?

**Published:** 1863-12

**Authors:** 


### WHAT ABOUT INDIA RUBBER?

Does India Rubber, alias Vulcanite, Coralite, etc., etc., injure the health of those wearing it, as a base for teeth?

Rubber for teeth is so nice,—no interstices for food to be retained in; it has been used for years; and besides, everybody likes it. It is of good report, and comes well-recommended.

Having never constructed a base of rubber for teeth, nor seen but few pieces of it, the writer of this pretends to be only a novice in regard to it. But, in common with many of his brethren, he remembers, with *regret*, gutta percha as a base for teeth, and the *depletion* suffered from its local peddlers of patent-rights. It was a wonderful improvement. A large dental furnishing establishment had the grand secret, unknown to any one else, of preparing the article at $8 per pound. But, two mischievous dentists of Cincinnati experimented and published, in the Dental Register, a mode of preparing the gutta percha, better than any ever offered in the market, at $3 per pound: when, as a base for teeth, it was soon numbered with the humbugs of the past.

Nor has he forgotten Cheoplasty, coming subsequently, of respectable parentage, rich in pretensions, a "new metal," that threw gold and platinum into outer darkness; with strong recommendations from some of the most respectable dentists of Baltimore, Philadelphia, and New York. In a few months, local agents were like vultures over the land, and a larger pamphlet came filled with certificates from the rank and file of the dental profession from the east, west, north, and south. What everybody said, must be true.

In its full tide of successful operation, when its lucky inventor and his agents were reaping the golden harvest, and the peans of the greatest of human benefactors resounded through the land, when not a word had been uttered against it by the dental press, Novice had the temerity to give Cheoplasty a gentle blow in the Dental Register; at which it gave one yelp, and was so dead it never moved again.

Perhaps the rubber may become a fixed institution in dental practice. But complaints are ominous; and failures of the holders of the patent right to prosecute at law those whom they know to be constantly infringing on their rights—so different from their former course in such cases—indicates a want of confidence in the ultimate success of the rubber as a base for teeth, or a want of confidence in the strength of the patient.

If it is supposed that rubber causes disease of the mouth, or is in any way injurious to the health of the patient, let us have the results of such cases reported. Let thorough tests of them be made public. If there are unfavorable indications from the use of rubber, let metal be worn until they disappear, and then the rubber again, to learn if they will return. Reports of well-conducted experiments will decide the question as to injury in some cases from the use of rubber; and if in the affirmative, the next thing required will be to ascertain the cause of the injury, which may be more difficult, as it is not probable it is by the coloring-matter in the rubber. NOVICE.

